# Effects of γ-Aminobutyric Acid on Growth Performance, Immunity, Antioxidant Capacity, and Intestinal Microbiota of Growing Minks

**DOI:** 10.3390/vetsci11090398

**Published:** 2024-08-29

**Authors:** Yalin Li, Shibo Zhen, Fengxue Sun, Lin Cao, Lihua Wang

**Affiliations:** College of Animal Science and Technology, Qingdao Agricultural University, Qingdao 266109, China; liyalin827@163.com (Y.L.); zhensbo@163.com (S.Z.); sunfengxue2022@163.com (F.S.); caolin19982024@163.com (L.C.)

**Keywords:** γ-aminobutyric acid, mink, growth performance, immunity, antioxidant capacity, intestinal microbiota

## Abstract

**Simple Summary:**

Previous studies in several species have demonstrated that dietary γ-aminobutyric acid (GABA) supplemented can improve the growth performance, immunity, and antioxidant activity of animals. However, the effects of GABA on growing minks are not well documented. Therefore, this study aimed to evaluate the effects of GABA on growth performance, immunity, and intestinal microbiota in growing minks, and to determine the optimal level of GABA supplementation for their development.

**Abstract:**

The present experiment was conducted to investigate the effects of γ-aminobutyric acid (GABA) on the growth performance, immunity, antioxidant capacity, and intestinal microbiota of growing minks. One hundred minks were evenly allocated across five groups, with each group consisting of 10 males and 10 females. The minks in these groups were fed a basal diet supplemented with γ-aminobutyric acid (GABA) at 0 (control), 10, 20, 30, and 40 mg/kg of diet, respectively. The experiment lasted for eight weeks. The results showed that GABA significantly affected immunity, antioxidant capacity, and intestinal microbiota (*p* < 0.05). Compared to the control minks, minks in 20, 30, and 40 mg/kg GABA group had greater total protein quantitative (TP), immunoglobulin A (IgA), immunoglobulin M (IgM) content, total antioxidant capacity (T-AOC), and glutathione peroxidase (GSH-Px) activities in serum as well as interleukin-4 (IL-4) level in jejunal mucosa (*p* < 0.05), and had less serum blood urea nitrogen (BUN) content (*p* < 0.05). Furthermore, compared with the control, the supplementation of GABA at 30 mg/kg of diet improved average daily feed intake (ADFI) (*p* < 0.05), increased immunoglobulin G (IgG) content in serum, interleukin-10 (IL-10) and secreted immunoglobulin A (SIgA) levels in jejunal mucosa, and decreased jejunal mucosal interleukin-2 (IL-2), interleukin-12 (IL-12), and interferon-γ (IFN-γ) levels (*p* < 0.05). The weight and feed intake of males were higher than females, and the feed/gain ratio (F/G) was lower than females (*p* < 0.05). Males also had greater serum superoxide dismutase (SOD) and GSH-Px activities, and jejunal mucosa IL-2, IL-4, IL-12, SIgA, and IFN-γ levels (*p* < 0.05), and males had less serum IgA, IgM, and T-AOC contents, and jejunal mucosal tumor necrosis factor-α (TNF-α) level (*p* < 0.05). The results suggest that the supplementation of GABA at 30 mg/kg of diet can improve immune status and antioxidant capacity, and modulate the intestinal microbiota abundance of growing minks.

## 1. Introduction

γ-aminobutyric acid(GABA), a non-protein amino acid with four carbon atoms [1], plays an essential role as a neurotransmitter in the mammalian nervous system [2]. While GABA is widely found in nature [2], its concentration in natural feed ingredients is typically insufficient to support optimal biological functions [3,4]. Therefore, dietary supplementation with exogenous GABA is necessary for animals to confer a range of health benefits. Zhang et al. [1] have demonstrated that GABA supplementation can enhance the performance and physiological condition of laying hens, and relieve heat stress through regulating hormone secretion, antioxidant and immune activities, and maintaining electrolyte balance. Wang et al. [5] discovered that dietary GABA supplementation improved feed intake, performance, and antioxidant status in perinatal dairy cows. Guang et al. [6] reported that the addition of GABA to the diet enhanced the growth performance of yellow-feathered broilers aged between 36 and 49 days under high-temperature conditions. This supplementation reduced serum intracellular enzyme activity, which contributed to the improved health of the broilers’ organs and the maintenance of the integrity of their intestinal morphology [6]. Mokhtar et al. [7] reported that GABA could regulate damage related to stress in broilers. Ngo et al. [2] proved that GABA decreased the production of pro-inflammatory mediators and ameliorated inflammatory symptoms, thereby inhibiting inflammation.

High ambient temperatures can reduce feed intake, feed conversion efficiency, and growth rates in livestock and poultry [8]. Mink, which naturally thrive in cold regions like North America and Northern Europe, prefer cooler climates over heat [9]. High ambient temperatures not only induce heat stress reactions in mink but also lead to a range of health problems, such as decreased appetite, weight loss, and reduced immune function [10,11]. Therefore, it is crucial to implement effective measures to ensure the health and productivity of mink under high-temperature environments.

**Figure 1:**
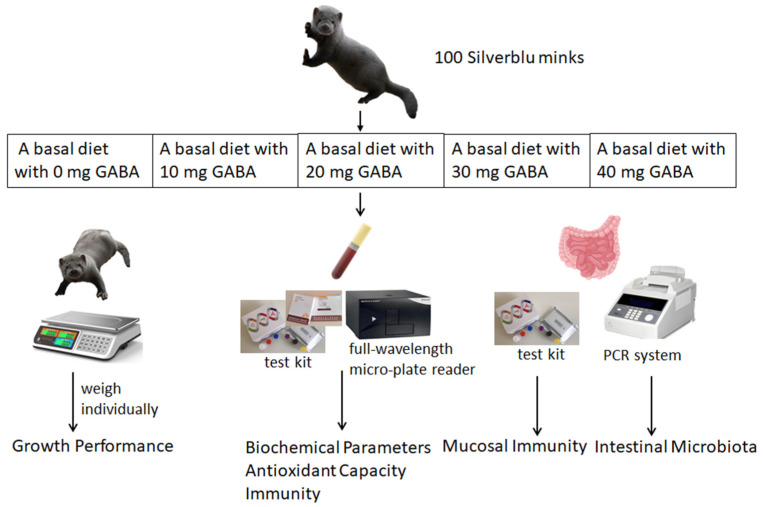
Experimental design drawing.

The increasing recognition of GABA’s potential as a feed additive has promoted extensive research into its application in the diets of poultry and ruminant animals [5,6,7]. However, the effects of GABA on growing minks are not well documented. This experiment aimed to assess the impact of GABA on growth performance, immunity, and intestinal microbiota in growing minks, and to determine the optimal level of GABA supplementation for their development (Figure 1).

## 2. Materials and Methods

### 2.1. Experimental Animals, Design and Management

The experiment was conducted at a commercial mink farm located in Haiyang, a region in the eastern part of China, from July to September in 2022. A total of 100 Silverblu minks, at approximately 12 weeks of age and with an average body weight of 1134.47 ± 22.76 g, were randomly selected from the farm’s animal stock for the study. All the minks were divided into five groups, each consisting of 10 male minks and 10 female minks. The minks in these groups were fed a basal diet supplemented with GABA at 0 (control), 10, 20, 30, and 40 mg of diet, respectively (Figure 1).

The GABA (99% purity) used in the present experiment was provided by Shanghai Macklin Biochemical Co., Ltd. (Shanghai, China). The experiment lasted for 8 wk after one week of adaptation. The diets used in the experiment were formulated according to the commercial standards. The composition of the diets (as-fed basis) and nutrient levels on an air-dry basis are detailed in Table 1.

All the minks were housed in an open-type barn with two rows of wire mesh cages. Each experimental mink was housed individually in a wire mesh cage. The cage included an attached straw-bedded nest box, which had an entrance for the mink to enter and exit.

Fresh mink diet was manufactured and provided by the farm twice a day (6:30 AM and 5:30 PM, respectively). Fresh water was supplied by a drinker in the cage. Both diet and water were available ad libitum during the experiment. The feed consumption and feces condition of the experimental minks were observed to evaluate the health of the minks daily throughout the study, and the death of the minks was recorded.

The ambient temperature was 25.53 ± 0.07 °C, relative humidity was 83.16 ± 0.24%, and the light schedule was a natural light regime throughout the study.

### 2.2. Sampling and Measurements

All the minks in the five groups were individually weighed at the beginning (week 0) and end (week 8) of the study to determine the initial and final body weights. The average daily gain (ADG) was then calculated. The apparent feed intake of the minks was monitored for three consecutive days (days 3 to 5) each week during the experiment. The given and remaining rations were weighed daily during the 3d monitoring period, respectively. Then, the average daily feed (ADFI) and feed/gain ratio (F/G) were calculated.

At the end of the study, six male and six female minks from each group were selected for blood collection via cardiac puncture. Following the blood collection, these minks were euthanized to obtain samples of the jejunal mucosal tissue and rectal content. The blood was centrifuged at 3000× *g* at 4 °C for 10 min, and subsequently, the serum was obtained. Jejunal mucosal tissue homogenate was prepared with 9 mL of 0.9% NaCl and 1 g of jejunal mucosal tissue. The tissue was centrifuged at 3500× *g* at 4 °C for 10 min, and the supernatant was obtained. The blood and the jejunal mucosal tissue supernatant were stored in a −20 °C refrigerator for further assessment, and the rectal content samples were stored in a −80 °C refrigerator for flora analysis.

### 2.3. Biochemical Parameters in Serum

The contents of albumin (ALB), blood urea nitrogen (BUN), and total protein quantitative (TP) in serum were determined by colorimetry. The absorbance of ALB, BUN, and TP was determined by a UV-VIS spectrophotometer (UV-6200, Shanghai, China). The activities of alanine aminotransferase (ALT) and aspartate aminotransferase (AST) in the serum were determined by the micro-plate method with a full-wavelength micro-plate reader. All these indicators were measured using kits manufactured by Jiancheng Biological Engineering Research Institute (Nanjing, China).

### 2.4. Immunity Analysis

The collected serum and jejunal mucosa samples were assayed for immune indices according to the methods of Li et al. [12]. The concentration of immunoglobulins (IgA, IgM, and IgG) in serum, and SIgA and cytokines (IL-4, IL-10, IL-2, IL-12, TNF-α, and IFN-γ) in jejunal mucosa were assayed using a commercial kit (Jiancheng Biological Engineering Research Institute, Nanjing, China) and read using a full-wavelength micro-plate reader.

### 2.5. Antioxidant Capacity Analysis

The serum total antioxidant capacity (T-AOC), activities of glutathione peroxidase (GSH-Px) and superoxide dismutase (SOD), and malondialdehyde (MDA) were quantitatively assessed using a colorimetric assay.

### 2.6. Intestinal Flora Analysis

The collected samples from the rectal contents were extracted DNA to determine intestinal flora according to the previous method [13]. The extracted genomic DNA from rectal contents was detected, amplified (ABI GeneAmp&reg, 9700, Applied Biosystems, Foster City, CA, USA), purified, recovered, quantified, and sequenced [14].

### 2.7. Statistical Analysis

The Proc Mixed model of the SAS 9.4 software (SAS Inst. Cary, NY, USA) was used to analyze the data of growth performance and, immunohistochemical and antioxidant capacity. The model included GABA, gender, and their interactions as fixed effects, and the cage as the experimental unit. The least square means were separated by Tukey adjustment. Differences were considered significant when *p* < 0.05.

The data of intestinal flora were analyzed on the I-Sanger cloud platform. The FLASH 1.2.11 software performs pair-end double-ended sequence splicing. The Spearman correlation coefficient was adopted to analyze the correlation of the intestinal flora with the immunity of minks.

## 3. Results

For the minks, no interactions for growth performance, biochemical parameters, immunity, intestinal microbiota, and antioxidant capacity (except ADFI and GSH-Px activities) between GABA and gender (all *p* > 0.05) were observed. Therefore, all the data are presented for main effects.

### 3.1. Growth Performance

GABA had significant effects on ADFI throughout the entire 8-week study period (*p* < 0.05; Table 2). However, these effects were not observed on the other performance metrics of the minks (*p* > 0.05). In comparison with the minks in the control, the minks in the groups fed diets supplemented with GABA at 10, 20, 30, or 40 mg/kg of diet had greater ADFI (*p* < 0.05). Male minks had greater initial BW, final BW, ADG, and ADFI, and less F/G than female minks (*p* < 0.05).

### 3.2. Biochemical Parameters in Serum

The effects of dietary GABA on the serum biochemical parameters of the minks are presented in Table 3. Compared to the control, GABA at 20, 30, and 40 mg/kg of diet increased TP and decreased BUN concentrations, while GABA at 10 mg/kg of diet decreased serum BUN concentration (*p* < 0.05). Males had lower ALT concentrations in serum than females (*p* < 0.05). 

### 3.3. Immune Function

#### 3.3.1. Serum Immune Indexes

Dietary GABA significantly increased the concentrations of IgA, IgG, and IgM in mink serum (*p* < 0.05; Table 4). Compared with the control, GABA at 20 and 40 mg/kg of diet significantly increased the serum IgA and IgM concentrations, while GABA at 30 mg/kg of diet increased significantly the serum concentrations of IgA, IgM, and IgG (*p* < 0.05). The male minks had less serum IgA and IgM concentrations than the females (*p* < 0.05). 

#### 3.3.2. Mucosal Immune Indexes

GABA affected the levels of SIgA, IL-4, IL-2, IL-10, IL-12, and IFN-γ in the jejunal mucosa (*p* < 0.05; Table 5). But there was no effect on the TNF-α level (*p* > 0.05). Compared to the control, GABA at 30 mg/kg increased SIgA, IL-4, and IL-10, and decreased IL-2, IL-12, and IFN-γ levels, while GABA at 20 mg/kg increased SIgA, IL-4, and IL-10 levels (*p* < 0.05), and GABA at 10 mg/kg increased IL-4 and IL-10 levels (*p* < 0.05). Males had greater SIgA, IL-4, IL-12, IL-2, and IFN-γ levels, and less TNF-α level in jejunal mucosal than females (*p* < 0.05). 

### 3.4. Antioxidant Capacity

GABA significantly affected the serum T-AOC and GSH-Px levels (*p* < 0.05; Table 6), but had no significant effects on the serum SOD activity and MDA concentration (*p* > 0.05). Compared with the control, GABA at 20, 30, and 40 mg/kg of diet enhanced the T-AOC and GSH-Px levels in serum (*p* < 0.05). The male minks exhibited greater SOD and GSH-Px activities, and less T-AOC levels compared to the female minks (*p* < 0.05).

### 3.5. Intestinal Microbiota

#### 3.5.1. Intestine Microbiota of Male Minks

**Figure 2:**
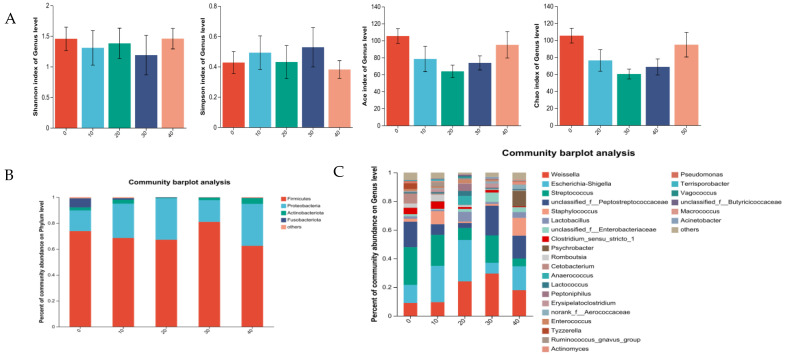
Effects of GABA on intestinal microbiota from rectal content in growing male minks. (**A**) Alpha diversity indices. (**B**) Mean relative abundance of phylum. (**C**) Mean relative abundance of genus.

GABA had no effect on Alpha diversity in the male minks (*p* > 0.05; Figure 2A). At the phylum level (Figure 2B), Firmicutes and Proteobacteria were dominant for the mink in all the groups. The data obtained confirmed that *Streptococcus*, *unclassified_f__ Peptostreptococcaceae*, *Escherichia-Shigella*, and *Weissella* were the most dominant genera for the males in the control (Figure 2C). *Escherichia-Shigella*, *Streptococcus*, *Weissella*, and *Staphylococcus* were dominant bacteria genera for the males in the group of 10 mg GABA/kg diet. *Escherichia*-*Shigella*, *Weissella*, *Streptococcus*, and *Lactobacillus* were dominant bacteria genera for the males in the group of 20 mg GABA/kg diet. *Weissella*, *unclassified_f__Peptostreptococcaceae*, *Streptococcus*, and *Escherichia*-*Shigella* were the dominant bacteria genera for the males in the group of 30 mg GABA/kg diet. *Weissella*, *Escherichia*-*Shigella*, *unclassified_f__Peptostreptococcaceae*, and *Staphylococcus* were the dominant bacteria genera for the males in the group of 40 mg GABA/kg diet. 

**Figure 3:**
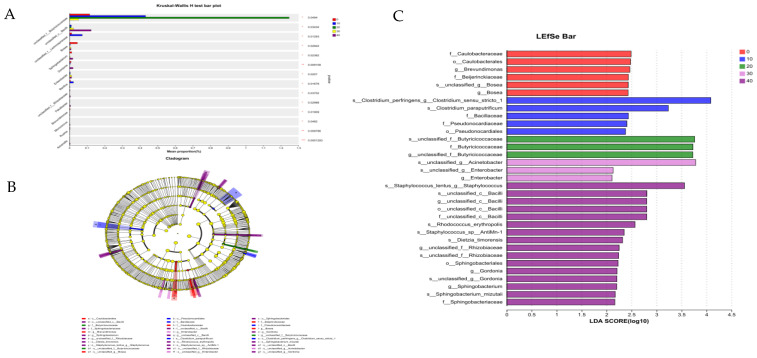
LEfSe analysis and significant changes in different rectal flora in the male minks at different levels. (**A**) A specific bacterium has been observed to manifest noticeable differences in the density of associated microbiological populations in the male minks. (**B**) The cladogram of the LEfSe analysis. (**C**) The LDA score (LDA = 2) generated by the LEfSe analysis; different colors indicate different groups. p_: phylum level; c_: class level; o_: order level; f_: family level; g_: genus level; s_: species.

GABA had significant effects on the relative abundances of *unclassified_f__Bacilli*, *unclassified_f__Lachnospiraceae*, *Enterobacter*, *Bosea*, *Sphingobacterium*, *Gordonia*, *Bacillus*, *unclassified_f__Rhizobiaceae*, *Patulibacter*, *Brevundimonas*, *Micrococcus*, *Ruania*, *Advenella* and *unclassified_f__Butyricicoccaceae* (*p* < 0.05; Figure 3A). In the gut of the males, 6 and 27 microorganisms at different taxonomic levels were identified in the control and the GABA supplemental groups, respectively, which could serve as potential biomarkers (Figure 3B). The linear discriminant analysis effect size (LEfSe) (LDA score = 2; Figure 3C) can determine the differences in the bacterial taxa between the control and the GABA supplemental groups. *Caulobacteraceae* (family), *Caulobacterales* (order), *Brevundimonas* (genus), *Beijerinckiaceae* (family), *Bosea* (genus), and *unclassified_f__Bosea* (species) were abundant in the control. *Clostridium_perfringens* (species), *Clostridium_paraputrificum* (species), *Bacillaceae* (family), *Pseudonocardiaceae* (family), and *Pseudonocardiales* (order) were more abundant in the group of GABA at 10 mg/kg of diet. *Butyricicoccaceae* (family), *unclassified_f__Butyricicoccaceae* (genus), and *unclassified_f__Butyricicoccaceae* (species) were more abundant in the group of GABA at 20 mg/kg of diet. *Enterobacter* (genus), *unclassified_f__Enterobacter* (species), and *unclassified_f__Acinetobacter* (species) were more abundant in the group of GABA at 30 mg/kg of diet. *Staphylococcus_lentus* (species), *Bacilli* (family), *Rhodococcus_erythropolis* (species), *Staphylococcus_sp__AntiMn-1* (species), *Dietzia_timorensis* (species), *Rhizobiaceae* (genus), *Sphingobacteriales* (order), *Gordonia* (genus), *Sphingobacterium* (genus), *Sphingobacterium_mizutaii* (species), and *Sphingobacteriaceae* (family) were more abundant in the group of GABA at 40 mg/kg of diet.

#### 3.5.2. Intestine Microbiota of Female Minks

**Figure 4:**
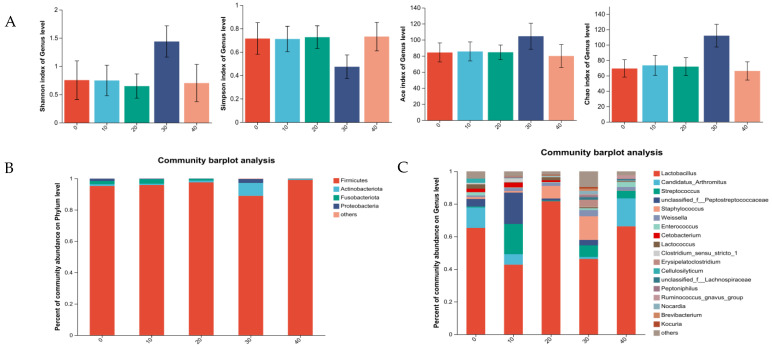
Effects of GABA on intestinal microbiota from rectal content in growing female minks. (**A**) Alpha diversity indices. (**B**) Mean relative abundance of phylum. (**C**) Mean relative abundance of genus.

GABA had no effect on Alpha diversity in the female minks (*p* > 0.05; Figure 4A). At the taxonomic phylum level (Figure 4B), the abundance of Firmicutes predominated across all the examined groups. The data obtained confirmed that *Lactobacillus*, *Candidatus*_*Arthromitus*, *unclassified*_*f__Peptostreptococcaceae*, and *Lactococcus* were the dominant bacteria genera for the females in the control (Figure 4C). *Lactobacillus*, *unclassified_f__Peptostreptococcaceae*, *Streptococcus*, and *Candidatus*_*Arthromitus* were the dominant bacteria genera for the females in the group of 10 mg GABA/kg diet. *Lactobacillus*, *Staphylococcus*, *Lactococcus*, and *Weissella* were the dominant bacteria genera for the females in the group of 20 mg GABA/kg diet. *Lactobacillus*, *Staphylococcus*, *Streptococcus*, and *Erysipelatoclostridium* were the dominant bacteria genera for the females in the group of 30 mg GABA/kg diet. *Lactobacillus*, *Candidatus*_*Arthromitus*, *Streptococcus*, and *Enterococcus* were the most abundant genera for the females in the group of 40 mg GABA/kg diet.

**Figure 5:**
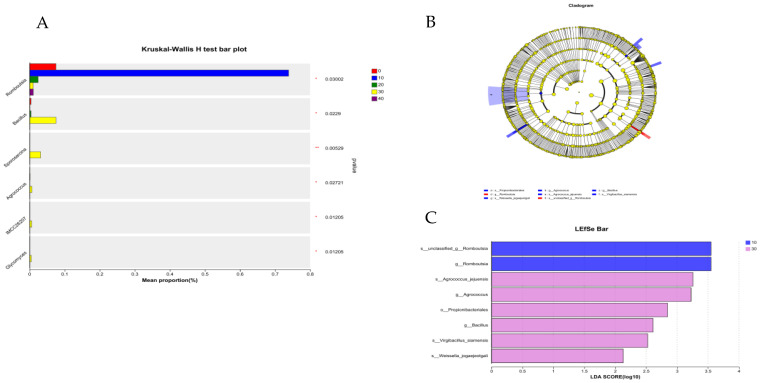
LEfSe analysis and significant changes in different rectal flora in female minks at different levels. (**A**) A species of bacteria exhibits variations in the abundance of microbial communities in the female minks. (**B**) The cladogram of the LEfSe analysis. (**C**) The LDA score (LDA = 2) generated by the LEfSe analysis; different colors indicate different groups. p_: phylum level; c_: class level; o_: order level; f_: family level; g_: genus level; s_: species.

GABA had significant effects on the relative abundance of *Bacillus*, *Sporosarcina*, *Agrococcus*, *IMCC26207*, *Glycomyces*, and *Romboutsia* (*p* < 0.05; Figure 5A). However, there were no significant differences in the microbiota abundance between the control and 20 mg/kg or 40 mg/kg GABA groups, respectively. In the gut of the females, 2 and 6 microorganisms at different taxonomic levels were identified in the 10 mg/kg GABA group and 30 mg/kg GABA group, respectively (Figure 5B). The linear discriminant analysis effect size (LEfSe) analysis (LDA score = 2) was used to identify the bacterial taxa with significant differences between the CON and GABA groups (Figure 5C). The relative abundances of *Romboutsia* (genus) and *unclassified_f__Romboutsia* (species) increased significantly in the 10 mg/kg GABA group, whereas *Agrococcus_jejuensis* (species), *Agrococcus* (genus), *Propionibacteriales* (order), *Bacillus* (genus), *Virgibacillus_siamensis* (species), and *Weissella*_*jogaejeotgali* (species) were more prevalent in the 30 mg/kg GABA group.

#### 3.5.3. Correlation Analysis

**Figure 6:**
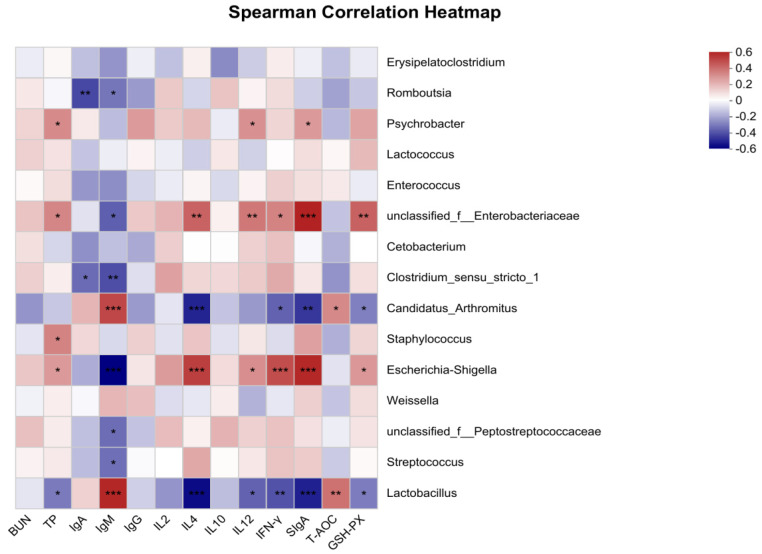
Heatmap of intestinal flora and immune correlation in minks (at genus level). * 0.01 < *p* ≤ 0.05, ** 0.001 < *p* ≤ 0.01, *** *p* ≤ 0.001.

The correlation analyses between the relative abundance of the dominant bacteria at the genus level and serum biochemical indices, immune indices, and serum antioxidant parameters of growing minks are presented in Figure 6. The serum TP concentration was positively associated with *Psychrobacter*, *Staphylococcus*, *Escherichia*-*Shigella*, and *unclassified_f__Enterobacteriaceae* (*p* < 0.05). Both serum IgA and IgM had negatively correlated with *Romboutsia* and *Clostridium*_*sensu*_*stricto*_*1*. *Unclassified_f__Peptostreptococcaceae* and *Streptococcus* had negatively correlated with serum IgM (*p* < 0.05). *Unclassified*_*f__Enterobacteriaceae* and *Escherichia-Shigella* had positively correlated with IL-4, IL-12, IFN-γ, and SIgA in jejunal mucosa and serum GSH-Px activity (*p* < 0.05), and negatively correlated with serum IgM (*p* < 0.05). *Candidatus*_*Arthromitus* had positively correlated with serum T-AOC and IgM (*p* < 0.05), and negatively correlated with IL-4, IFN-γ, SIgA, and GSH-Px (*p* < 0.05). Interestingly, *Psychrobacter* had positively correlated with SIgA and IL-12 (*p* < 0.05), while *Lactobacillus* was positively associated with IgM and T-AOC (*p* < 0.05), and negatively associated with TP, IL-4, IL-12, IFN-γ, SIgA, and GSH-Px (*p* < 0.05).

## 4. Discussion 

The results of the experiment indicated that GABA had no effects on BW and ADG of growing minks. This is the same opinion as the consequences of the previous studies on pigs that GABA had no effects on the BW and ADG of growing-finishing pigs [15]. However, the supplementation of GABA at 10, 20, 30, or 40 mg/kg of diet increased the feed intake of minks. Especially in the case of high temperatures, the increase in feed intake can mitigate the suppressive action of heat stress in terms of feed intake. As suggested by Jonaidi et al. [16], GABA stimulated feed intake by interaction with neuropeptide Y (NPY). In addition, GABA is an important inhibitory neurotransmitter and sedative, which agents in the central nervous system and can improve the feed intake and absorption of animals by regulating feed intake center, promoting gastric acid secretion, inhibiting cholecystokinin secretion, and suppressing stomach satiety center [1].

The present study proved that GABA at 20, 30, and 40 mg/kg of diet increased TP, and GABA at 10, 20, 30, and 40 mg/kg of diet decreased the BUN concentrations. Additionally, GABA tended to decrease the AST activity in serum. These findings are consistent with the results reported by Ncho et al. on chickens [17]. GABA inhibits glutamic acid decarboxylation by reducing the activities of alkaline phosphatase and aminotransferase, and reacts with α-ketoglutaric acid to produce glutamic acid, thereby reducing the content of nitrogen in serum, subsequently reducing the content of nitrogen excreted in urine [18], and promoting the deposit of protein. According to Zhang et al. [19], TP is the combination of a range of proteins, which is synthesized by the liver. In addition, AST as an intracellular enzyme is determined to assess liver necrosis. The elevation of AST in serum indicates that AST is released into the blood by the damaged hepatocyte [20]. Both the reduction in the AST activity and the elevation in the TP concentration in serum are associated with the better functioning of the liver [17]. Thus, the present study suggested that GABA supplementation is beneficial to liver function.

The experimental results confirmed that supplemental GABA at 20, 30, and 40 mg/kg of diet increased the serum IgA and IgM concentrations, and GABA at 30 mg/kg of diet enhanced the IgG concentrations in serum. Several studies proved that GABA could improve the concentrations of IgA, IgM, and IgG [21,22]. IgM is mediated by the activation of the complement system as a defense against foreign substances. IgA drives mucosal responses against pathogens. IgG is involved in secondary reactions against toxins and viruses [23]. GABA promotes T lymphocyte proliferation and reduces macrophage apoptosis by inhibiting somatostatin and adrenal corticosteroid hormone secretion [24,25,26], thereby enhancing immunoglobulin production and improving the humoral immunity of the body.

The first line of defense against any pathogens in the intestine is the intestinal immune system [27]. In this study, the minks in the GABA groups had greater levels of IL-4 and IL-10 in the mucosa. The minks in the 30 mg/kg GABA group had lower levels of mucosal IL-12 and IFN-γ. Additionally, the minks in the 20 and 30 mg/kg GABA groups had higher levels of SIgA. The findings suggest that 30 mg/kg GABA is more effective in modulating intestinal immunity. As suggested by Chen et al. [28], dietary GABA promoted the gene expression of SIgA, IL-4, and IL-10 in weaning piglets. Intestinal mucosa can express inflammatory cytokines produced by the intestinal immune system, SIgA produced by plasma cells, and other immune factors [29,30]. SIgA is the effector component of the mucosal immune system [31], and promotes SIgA secretion by promoting polymeric immunoglobulin receptor (pIgR) expression [27,32]. Tanga et al. [33] observed that a decrease in TLR4 leads to a decrease in pro-inflammatory cytokine expressions such as IL-12 and IFN-γ. GABA may suppress the expression of the TLR4/NF-κB pathway and inhibit the activation of TLR4 by activating GABARs, thus affecting downstream inflammatory processes [15]. Multiple studies proved that IL-10 signaling plays a positive role in the maintenance of appropriate epithelial permeability in the gut epithelium [34,35,36]. In addition, GABA regulates T cell proliferation and promotes IL-10 production [36,37,38]. It is probable that GABA modulates the expression of immune factors by triggering M1 and M2 immune responses and signaling pathways (IRFs, STATs, NF-κB, and SOCS) [39].

In agreement with previous studies [15,40], dietary GABA at 20, 30, and 40 mg/kg of diet increased the activities of T-AOD and GSH-Px. T-AOC is a critical indicator of the functional status of the body’s antioxidant system [41]. GSH-Px catalyzes the reduction in H2O2 and lipid peroxides [42]. The exposure of the gut to foreign substances and microbial pathogens produces ROS, and the excessive production of ROS can affect intestinal health, resulting in various intestinal diseases [43]. Dietary GABA supplementation can enhance antioxidant enzymes and the activities of GSH, thereby reducing ROS production and lipid peroxidation [1]. GABA is involved in reducing the production of ROS by inhibiting the expression of the enzymes involved in NF-κB signaling, thereby blocking the occurrence of inflammatory reactions, and improving the antioxidant capacity of animals under stress [15,43].

The study demonstrated that GABA markedly modulated the compositions and diversity of intestinal flora. It suggests that GABA performs an essential function in preserving intestinal microbial homeostasis. The comparative analysis revealed a distinct relative abundance of the microbiota between the male and female subjects at both the phylum and genus taxonomic levels. However, Firmicutes was the most dominant in both the males and females. This finding is consistent with the study by Bahl et al. on mink colon microbiota [44]. Most of the bacteria in the phylum Firmicutes are beneficial [45]. At the genus level, the relative abundance of *Weissella* and *Streptococcus* increased, while that of *Lactobacillus* and *Escherichia-Shigella* decreased, which differs from the results reported by Chen et al. [27]. This discrepancy may be due to minks being different from other domestic animals. The genus *Weissella* has probiotic properties and biotechnological potential [46]. *Clostridium sensustricto* (*cluster I*) is a type of bacteria capable of using glucose to produce short-chain fatty acids [47]. *Advenella* is a potential degrader of organic pollutants [48]. GABA effectively lowers gut pH by increasing the concentrations of short-chain fatty acids, particularly propionic and butyric acids, and consequently suppresses the proliferation of pathogenic bacteria [49,50].

Previous studies have noted that GABA could potentially influence host metabolism through the modulation of the gut microbiota [51,52]. The supplementation of GABA has been shown to modulate the gut microbiota in ETEC-infected piglets by promoting the richness and diversity of the microbial community [27]. Our study investigated the interaction between the intestinal microbiota genera and gut immune indicators. We discovered a positive association between the genus *Psychrobacter* and SIgA and IL-12. *Psychrobacter* has potential antibiofilm properties [53]. Previous research has indicated that *Psychrobacter* possesses the ability to modulate immune system function, bolster antioxidant capacity, and augment disease resistance, thereby exerting a prebiotic effect on the health of exopalaemon carinicauda [54]. We also discovered that *Lactobacillus* was positively associated with IgM and T-AOC, but negatively associated with IFN-γ, SIgA, and GSH-Px. These findings suggest that *Lactobacillus* may not contribute positively to mink health. These results indicate that GABA can be used as a regulator of intestinal microbiota to mediate metabolism and the immune system.

## 5. Conclusions

The study’s findings show that GABA improves immune status and antioxidant capacity, and modulates the intestinal microbiota abundance in growing minks. Particularly, dietary supplementation with 30 mg/kg of GABA is more effective in modulating immunity compared to supplementation with 10, 20, and 40 mg/kg.

## Figures and Tables

**Table 1:** Ingredient composition (as-fed basis) and chemical composition (air-dry basis) of basal diets.

Items	Content (%)
Sea fishes	30.00
Unhatched fertilized egg	24.00
Chicken ribs	10.00
Chicken head	15.00
Extruded corn	7.00
Chicken livers	5.00
Wheat bran	3.00
Porcine spray-dried blood cells	2.00
Soybean meal	3.00
Premix ^1^	1.00
Total	100.00
Nutrient levels	
ME (MJ/kg) ^2^	16.59
Ether extract	20.30
Crude protein	33.55
Calcium	0.90
Phosphorus	0.97

DM = dry matter; ME = metabolic energy. ^1^ the premix provided the following per kg of the diets: nicotinic acid 20 mg, pantothenic acid 6 mg, folic acid 0.5 mg, biotin 0.5 mg, VA 9000 IU, VC 40 mg, VE 20 mg, VK_3_ 0.5 mg, VB_1_ 5 mg, VB_2_ 3 mg, VB_6_ 2.5 mg, VB_12_ 1 mg, VD_3_ 2000 IU, Fe 30 mg, Zn 25 mg, Mn 10 mg, Cu 5 mg, I 0.25 mg, and Se 0.2 mg. ^2^ the metabolizable energy (ME) is the calculated value, whereas the other values represent the actual measurements.

**Table 2:** Effects of GABA on growth performance of growing mink.

	GABA Supplemental Levels/(mg/kg)					SEM	Gender		SEM	*p*-Value		
	0	10	20	30	40		Male	Female		*P*_GABA_	*P*_Gender_	*P*_GABA×Gender_
Wt/g												
Initial (wk 0)	1130.50	1100.15	1132.12	1148.42	1124.14	32.59	1308.53	945.60	19.86	0.880	<0.001	0.967
Final (wk 8)	1835.25	1837.25	1908.74	1969.01	1936.89	55.55	2383.21	1411.64	33.85	0.327	<0.001	0.225
ADG, g	12.59	13.16	13.87	14.65	14.51	0.75	19.19	8.32	0.49	0.288	<0.001	0.100
ADFI, g	271.36 ^b^	283.55 ^a^	294.58 ^a^	288.64 ^a^	294.14 ^a^	5.80	349.50	223.41	3.75	0.044	<0.001	0.033
F:G	23.92	24.94	23.88	21.96	24.01	1.26	19.07	28.42	0.82	0.640	<0.001	0.407

Wt = weight; ADG = average daily gain; ADFI = average daily feed intake; F:G = feed/gain ratio. Means within a row and main effect with unlike superscripts differ (*p* < 0.05).

**Table 3:** Effects of GABA on biochemical indices of growing mink.

	GABA Supplemental Levels/(mg/kg)					SEM	Gender		SEM	*p*-Value		
	0	10	20	30	40		Male	Female		*P*_GABA_	*P*_Gender_	*P*_GABA×Gender_
ALB, g/L	46.35	46.23	51.48	51.00	49.69	1.82	47.36	50.54	1.15	0.131	0.058	0.780
BUN, mmol/L	10.44 ^a^	6.48 ^b^	6.05 ^b^	6.62 ^b^	7.58 ^b^	0.84	7.51	7.35	0.54	0.006	0.833	0.474
TP, g/L	33.07 ^c^	35.46 ^bc^	41.88 ^ab^	47.93 ^a^	42.60 ^ab^	2.65	42.27	38.11	1.68	0.002	0.089	0.087
AST, U/L	91.55	74.90	80.73	55.57	51.33	10.92	71.14	70.49	6.91	0.062	0.948	0.282
ALT, U/L	63.55	56.39	68.78	48.61	74.21	9.91	52.92	71.70	6.27	0.401	0.041	0.455

ALB = albumin; BUN = blood urea nitrogen; TP = total protein quantitative; AST = aspartate aminotransferase; ALT = alanine aminotransferase. Means within a row and main effect with unlike superscripts differ (*p* < 0.05).

**Table 4:** Effects of GABA on serum immune indices of growing mink.

	GABA Supplemental Levels/(mg/kg)					SEM	Gender		SEM	*p*-Value		
	0	10	20	30	40		Male	Female		*P*_GABA_	*P*_Gender_	*P*_GABA×Gender_
IgA, g/L	0.34 ^c^	0.31 ^c^	0.38 ^b^	0.54 ^a^	0.40 ^b^	0.01	0.38	0.42	0.01	<0.001	<0.001	0.195
IgM, g/L	2.87 ^c^	3.31 ^bc^	3.67 ^ab^	4.05 ^a^	3.44 ^b^	0.17	2.93	4.01	0.11	<0.001	<0.001	0.980
IgG, g/L	18.50 ^bc^	11.33 ^d^	15.51 ^cd^	25.46 ^a^	23.60 ^ab^	2.00	20.20	17.56	1.26	<0.001	0.147	0.192

IgA = immunoglobulin A; IgM = immunoglobulin M; IgG = immunoglobulin G. Means within a row and main effect with unlike superscripts differ (*p* < 0.05).

**Table 5:** Effects of GABA on mucosal immune indices of growing mink.

	GABA Supplemental Levels/(mg/kg)					SEM	Gender		SEM	*p*-Value		
	0	10	20	30	40		Male	Female		*P*_GABA_	*P*_Gender_	*P*_GABA×Gender_
IL-2, pg/mL	212.31 ^a^	194.50 ^ab^	190.90 ^ab^	173.07 ^b^	196.29 ^ab^	8.64	203.59	183.24	5.47	0.045	0.011	0.278
IL-4, pg/mL	98.75 ^c^	110.42 ^ab^	118.80 ^a^	116.88 ^ab^	108.97 ^b^	2.99	119.93	101.60	1.89	<0.001	<0.001	0.428
IL-10, pg/mL	56.83 ^b^	66.65 ^a^	69.07 ^a^	67.43 ^a^	63.58 ^ab^	2.71	66.55	62.88	1.71	0.021	0.136	0.364
IL-12, pg/mL	15.74 ^a^	15.28 ^a^	13.85 ^ab^	11.62 ^b^	15.29 ^a^	0.88	15.58	13.14	0.56	0.015	0.004	0.135
IFN-γ, pg/mL	605.40 ^a^	511.46 ^ab^	527.87 ^a^	405.02 ^b^	565.79 ^a^	38.64	592.09	454.13	24.44	0.009	<0.001	0.592
TNF-α, pg/mL	382.72	367.05	324.52	343.07	365.12	25.16	269.96	443.03	15.91	0.519	<0.001	0.872
SIgA, ng/mL	1672.03 ^b^	1861.26 ^b^	2310.15 ^a^	2363.17 ^a^	1805.48 ^b^	123.99	2429.79	1575.05	78.42	<0.001	<0.001	0.104

IL-2 = interleukin-2; IL-4 = interleukin-4; IL-10 = interleukin-10; IL-12 = interleukin-12; IFN-γ = interferon-γ; TNF-α = tumor necrosis factor-α; SIgA = secreted immunoglobulin A. Means within a row and main effect with unlike superscripts differ (*p* < 0.05).

**Table 6:** Effects of GABA on antioxidant capacity of growing mink.

	GABA Supplemental Levels/(mg/kg)					SEM	Gender		SEM	*p*-Value		
	0	10	20	30	40		Male	Female		*P*_GABA_	*P*_Gender_	*P*_GABA×Gender_
SOD, U/mL	60.61	52.94	56.59	71.40	57.36	5.74	73.21	46.35	3.63	0.214	<0.001	0.170
T-AOC, U/mL	12.46 ^b^	10.55 ^b^	20.11 ^a^	19.67 ^a^	18.41 ^a^	2.05	13.29	19.18	1.30	0.003	0.003	0.069
MDA, nmol/mL	8.23	7.73	7.94	14.42	8.90	2.25	8.88	10.01	1.42	0.198	0.578	0.687
GSH-Px, U/mL	828.89 ^c^	889.07 ^bc^	948.11 ^ab^	1017.20 ^a^	1056.99 ^a^	39.77	1048.56	847.55	25.15	0.001	<0.001	0.019

SOD = superoxide dismutase; T-AOC = total antioxidant capacity; MDA = malondialdehyde; GSH-Px = glutathione peroxidase. Means within a row and main effect with unlike superscripts differ (*p* < 0.05).

## Data Availability

None of these data are stored in an official repository. Support obtaining study data from authors upon request.
